# Instructional Programs Influencing the Enhancement of the Knowledge Required by Volunteers in Disasters: A Systematic Review

**DOI:** 10.30476/BEAT.2021.89340.1230

**Published:** 2021-07

**Authors:** Milad Ahmadi Marzaleh, Rita Rezaee, Mahmoudreza Peyravi

**Affiliations:** 1 *Research Center for Emergency and Disaster Resilience, Red Crescent society of the Islamic Republic of Iran, Tehran; Student Research Committee, Department of Health in Disasters and Emergencies, Health Human Resources Research Center, School of Management and Medical Informatics, Shiraz University of Medical Sciences, Shiraz, Iran*; 2 *Health Human Resource Development Research Center, Department of Health Information Management, Health Human Resources Research Center, School of Management and Medical Informatics, Shiraz University of Medical Sciences, Shiraz, Iran.*; 3 *Department of Health in Disasters and Emergencies, Health Human Resources Research Center, School of Management and Medical Informatics, Shiraz University of Medical Sciences, Shiraz, Iran*

**Keywords:** Volunteers, Disaster, Planning, Awareness, Knowledge.

## Abstract

**Objective:**

To determine the instructional programs required by volunteers based on the studies carried out worldwide.

**Methods:**

A systematic search was carried out by PubMed, Cochran Library, Scopus, EMBASE, Science Direct, Web of Science and ProQuest databases between January 1970 and the end of June 2019. The articles were selected based on the keywords chosen by the author. In the end, the volunteer’s instructional titles were extracted from the articles in disasters.

**Results:**

Eleven articles were chosen for final analysis after studying the titles, abstracts, and complete articles texts which 45 instructional titles were extracted. The most frequent scales in terms of repetition were ethics, kinds of exercises, personal protection instruments, general hygiene, awareness of certain disasters, accident command system, disaster triage and emergency planning.

**Conclusion:**

Governments should offer programs that can best serve the improvement of their performance by considering the daily increasing growth in the number of volunteers and in natural and manmade disasters. Universities and schools play determinant roles in this regard. It is hoped that the present study findings can be effective in codify an efficient instructional program for elevating the performance of the volunteers by taking part in disasters response.

## Introduction

People who lives in developing countries are usually more influenced by natural disasters. In fact, the majority of mortalities and casualties occur in these countries [1]. Human beings suffer from natural and manmade disasters because these accidents cause homelessness, food shortage, outbreak of diseases, and emergence of psychological and mental ailments [2]. Natural disasters leave damages behind more in developing countries since there is no comprehensive plan to respond an emergency condition in these countries [3, 4]. Various groups usually engage in relief and rescue operations as volunteers in natural and manmade disasters.

Volunteer refers to an individual who tends to spend time for a good deed without expecting any money in exchange [5]. Volunteers are expected to prove a more accentuated presence in disasters’ management and their risks reduction in international levels [6]. Volunteers are expected to exhibit a more highlighted presence and role in the future disasters according to the enhancing need of the society’s resilience. However, the volunteer’s role has undergone notable changes in the 21^st^ century [7]. Volunteers play vital roles in responding and monitoring during disasters. For example, volunteers were actively participated in preparation of risk maps for flood-prone regions in Queensland State of Australia between 2010 and 2011 [8]. Digitalization of the volunteers’ presence and activity is also a great importance in disaster management by considering the modern technologies growth in the contemporary world [9, 10]. Volunteers’ activities might last from days to months for disaster management [11]. Volunteers can obtain fast information regarding flood through local environments. The information prepared by individuals is readily discernable by the volunteers who can provide managers with precise and reliable information [12].

Volunteers are recommended to be trained; therefore, their capacities can be put into practical use at the time of disasters when there are few or no professionals [13]. The relevant instructional subjects in regard to save drowned persons are including Cardiopulmonary resuscitation (CPR), injury and fracture management and emergency bleeding control. The instructional needs should be matched with the organization vista and mission as well as with the local government’s support. They should be adjusted to the local values, rites, and customs as well [14]. The disaster-responding volunteers might have special psychological health needs. Thus, certain interventions have to be taken into account for psychologically supporting the volunteers who have stress and worries and are not mentally prepared [15].

Overall, the majority of countries around the globe are disaster-prone. Volunteers pervasively and comprehensively attend various disaster scenes but they usually have relatively low knowledge and awareness. The present study aims to determine the instructional programs required by volunteers based on the studies that have been carried out worldwide.

## Materials and Methods

*Eligibility Criteria and Search Strategy*

The study protocol was first registered in PROSPERO database with the identification number CRD42020187680. In this systematic review, the search method was based on the Preferred Reporting Items for Systematic Reviews and Meta-Analysis (PRISMA). A systematic search was conducted in peer-reviewed and English texts from January 1970 to June 2019 which were related to the study question; i.e., “what instructional programs are required to enhance the volunteers’ knowledge and awareness in disasters?”. At first, a general and swift search was carried out in Cochrane Library Database to ensure that no systematic reviews were carried out in this regard. No similar articles were found. Then, searches were conducted in electronic databases like PubMed, Cochran Library, Scopus, EMBASE, Science Direct, Web of Science, and ProQuest. Moreover, additional searches were made in grey literature includes books, internet websites, conference articles and dissertations. The group of using words was made the operator “and”, and it was considered as separate concepts. Furthermore, operator “or” was applied between the synonymous words. Searches were conducted in the titles, abstracts, and keyword of the articles. In PubMed database, MESH term was utilized for finding the articles. The employed search strategy has been shown in Table 1. Since there was no comparison group in this study, “C” or the very comparison group was not taken into consideration in population, intervention, comparison and outcomes (PICO). The search keywords were selected by the researcher. In the end, the factors were extracted from the selected articles. In the next stage, a complete list of the references was prepared from all the articles and the articles’ titles were investigated by the researchers. Accordingly, the articles irrelevant to the study goal were eliminated. For more assurance, all search stages were replicated twice. Endnote, version X8.1 was the software of choice for managing the resources.

**Table 1:** The search strategies used based on the instructional programs for enhancing the volunteers’ knowledge and awareness in the course of disasters

**PICO**	**#1 AND #2 AND #3**	**Strategy**
P	Volunteer OR Candidate OR Applicant OR Voluntary Worker	#1
I	Disaster OR Catastrophe OR Emergency OR Event OR Accident OR Incident OR Hazard OR Natural Disaster OR Flood OR Earthquake OR Hurricane OR Cyclone OR Tornado OR Volcanic Eruption OR Manmade Disaster	#2
O	Education OR Literacy OR Awareness OR Knowledge OR Wisdom OR Cognition OR Information	#3

Inclusion Criteria

Firstly, the keywords related to instructional programs required by the volunteers in disasters had to exist in the titles, abstracts and keywords of the searched articles. The related works’ abstracts were studied and finally the whole articles were analyzed by using assessment tools. The searches in the present systematic review were carried out for the articles published from 1970 to the end of June 2019. Unpublished articles (grey literature), protocols, conference articles, guidelines, instruction manuals, and credible organizations’ reports were explored, as well. The review, qualitative and quantitative articles were selected. Peer-reviewed articles were also selected.

Exclusion Criteria

The articles of irrelevant variables to the study question were omitted.

Screening

At first, all articles title were investigated by the author from the databases. The articles that met the inclusion criteria and were related to the study question were chosen. In the next stage, the selected articles’ abstracts were read by the author. Next, the parallel articles of the study goal and inclusion criteria were selected and the articles perfect texts were read and evaluated by the author. In the end, the instructional titles’ articles required by the disaster’s volunteers were selected. PRISMA guidelines were employed to evaluate the articles. Documentation and publication biases were also taken into consideration and the articles with a high rate of documentation were carefully evaluated. Quantitative articles were evaluated with strengthening the reporting of observational studies in epidemiology (STROBE) checklist, qualitative articles with consolidated criteria for reporting qualitative research (COREQ) checklist, mixed methods articles with consensus-based Standards for the selection of health status Measurement Instruments (COSMIN) checklist and review articles with PRISMA checklist. The entire aforementioned stages were repeated twice.

Data Extraction

The required information was extracted based on a summarization and data gathering form after carefully reading the articles. The information includes the corresponding author, study population, study sample size, study period, study design, data gathering instrument, study method, results, limitations, conclusion and instructional titles required by the volunteers. The summarization forms were completed for each of the selected articles. Two researchers completed all the forms and exhibited their tabular form following the investigation of all the articles. Finally, other study’s researchers offered their ideas regarding the conflict issues in the articles. The forms were composed in Microsoft Word, version 2016.

## Results

**Fig. 1:**
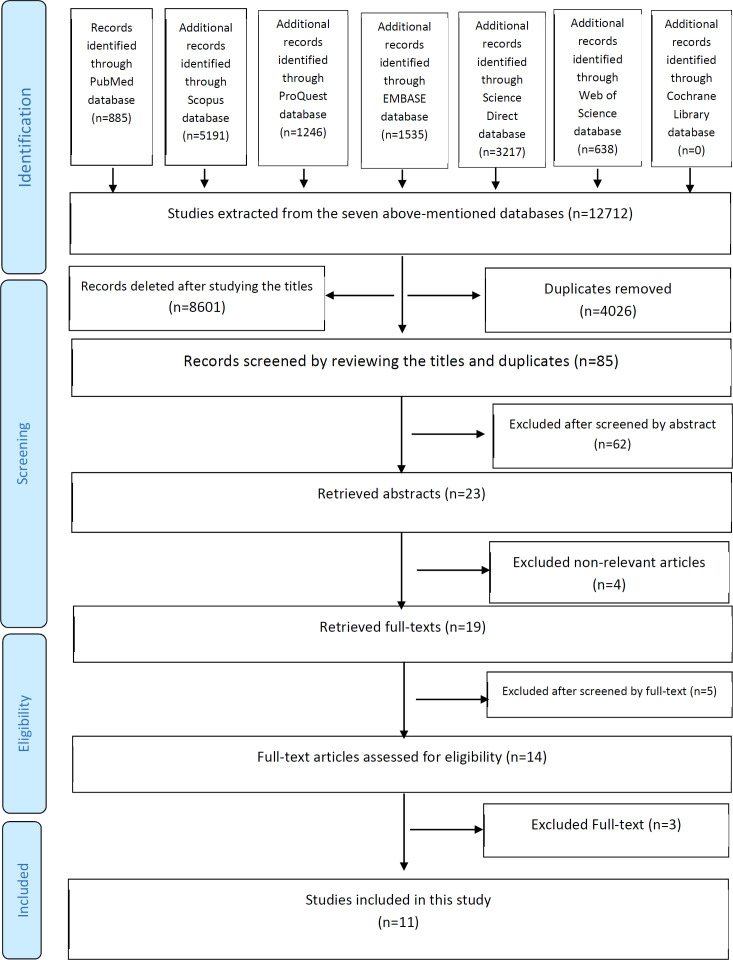
PRISMA flow diagram for the systematic review process

After searching the databases, 12712 articles were selected. Of these, 4026 were omitted because they were found repeated in various databases. After investigating 8686 article titles, 8601 were excluded because they were found not to be consistent with the study goal. The 85 remained articles’ abstracts from the previous stage were studied and 71 articles were excluded due to not being relevant to the study goal. Eventually, 14 complete article texts were selected for this study. In the end, 11 complete articles’ texts were found to be perfectly matched with the study goal. The articles selection process has been depicted in Figure 1.

A summary type of the extracted articles has showed in Table 2. Accordingly, two articles included a mixed method research, six articles were quantitative and three articles were review studies.

**Table 2:** The type of the selected articles

**Study type**	**Resources **	**Percentage (%)**	**Number**
Quantitative	[16-21]	54.54	6
Review	[14, 22]	18.18	3
Mixed method	[7, 13, 15]	27.27	2

The results analysis obtained from the selected articles has showed in Table 3. After the final analysis, 44 instructional titles were extracted. The most frequent criteria of repetition were ethics, exercises’ different types, personal protection instruments, general hygiene, awareness of special disasters, accident command system, disaster triage, emergency planning and disaster triage.

**Table 3:** Categories and sub-categories of the instructional titles required by the volunteers in disasters

**Categories**	**Sub-categories**	**Frequency**	**References**
Disaster Medicine issues	Lifeguarding	1	[14]
	CPR	1	[14]
	Management of special wounds	1	[14]
	Emergency bleeding control	1	[14]
	Disaster triage	2	[13, 18]
	Decontamination	1	[18]
	Child care	1	[20]
	Adults’ care	1	[20]
Public health issues	Public health	2	[17, 19]
	Epidemiology/surveillance system	1	[19]
	Psychological health	1	[15]
	Psychological first aid (hear, protect, and join)	1	[22]
	Psychological reactions in children	1	[22]
	Adults’ psychological response to disasters	1	[22]
Disaster management issues	Logical decision-making	1	[16]
	Various kinds of exercises	2	[17, 21]
	Personal protection equipment (PPE)	2	[17, 18]
	Awareness of special disasters (CBRNE incidents)	2	[17, 18]
	Incident command system (ICS)	2	[17, 18]
	Lingual skills (multilingualism)	1	[17]
	Population control and management	1	[17]
	Risk relationships	1	[17]
	Disaster terminology	1	[18]
	Emergency planning	2	[18, 19]
	Surge capacity	1	[18]
	Plans for taking measures in the course of accidents	1	[18]
	Ability to use guidelines	1	[18]
	Communications	2	[13, 19]
	Information management	1	[19]
	Motivation	1	[20]
	Regional monitoring	1	[19]
	Skills in establishing camps and shelters	1	[20]
	Leadership	1	[20]
	Emergency evacuation	1	[21]
	Early warning system	1	[13]
	Search and rescue	1	[13]
	Logistics	1	[13]
	Organizing activities	1	[13]
	National and local cultures	1	[7]
	Disaster effects	1	[22]
	Identification of high-risk persons in disasters	1	[22]
	Use of modern technologies	1	[7]
	Ethics	2	[16, 18]
	Safety kits	1	[18]

After reading the perfect texts of the 11 articles, the results were summarized in Table 4. The information includes title, corresponding author, study population, sample size, country, accomplishment time, data gathering instrument, method, results, limitations, conclusion, and instructional title (Table 4). 

**Table 4:** The included studies for final analysis

**No. **	**Corresponding author**	**Study goal**	**Study population**	**Sample size**	**Country**	**Study time**	**Study design**	**Data gathering tools**	**Study method**	**Results**	**Limitations**	**Conclusion**	**Instructional title**
1	Levina Chandra Khoe	Specifying the instructional needs and evaluating the general hygiene instructional program in disasters based on the evidence in Guangxi volunteers	NGOs and Chinese volunteers	Four NGOs were interviewed. 31 volunteers participated in first aid instruction.	China	2018	Mixed method	Interview and questionnaire before and after	The first stage was a qualitative research wherein telephone and skype interviews were made with the local NGOs’ volunteers. In the second stage, the NGOs’ members were invited to an instructional session. The instructional program was evaluated by a given pretest and posttest. In the end, an online questionnaire was administered to the participants for determining the instructional needs.	The results of the interview with the NGOs indicated that they emphasized that the instructions should be aligned with the organization’s panorama. The mean score was 8.04 before the instruction and 11.09 after that. Saving the drowning persons, CPR, injury management, and emergency bleeding control were the most frequently repeated instructional subjects as pointed out by the participants.	Not mentioned	The instructional needs have to be in line with the organization’s vista and mission, local government, and background and local values and customs.	Emergency bleeding control Lifeguarding CPR Injury management
2	Didin Wahyudin	This study aimed at developing mobile games for learning with the objective of developing ethical decision-making skills in disasters.	High schools and universities in Indonesia	High schools and novice universities in Indonesia	Indonesia	2013	Cross-sectional	Designing questionnaires and instructional programs	In the first stage, the awareness levels of novice volunteers in high schools and universities were evaluated in terms of their ethical decision-making in disasters using a questionnaire. Questions were designed in six subsets, namely 1) hugeness of impact, 2) social outcome, 3) impact contingencies, 4) emergent interventions, 5) consistency, and 6) impact rate. The instructional program was designed based on this questionnaire.	The results of the instructions showed that the instructional program was necessary for the volunteers.	Not mentioned	The more the relevant instructions are increased with the elevation of awareness of logical decision-making in disasters, the more the volunteers’ performance will be improved and enhanced.	Ethical issues Logical decision-making
3	R.J. Emery	It aimed at assessment of the hospital and surge capacity volunteers’ preparedness in hospitals in responding to the scenario.	US hospitals’ volunteers.	100	USA	2009	Cross-sectional	Physician volunteers in Texas Hospital	Implementation of the scenario of a large number of population’s contamination with radioactive materials	The radioactive incident scenario was executed in one of the US’s hospitals and the hospital volunteers responded to this accident and their performance was evaluated.	The results indicated that the extreme congestion of the hospital had a positive effect on the volunteers’ understanding and perception of their roles in disasters.	It has to be taken into consideration in the organizations’ strategic planning that holding of instructional and exercising sessions helps increase the volunteers’ preparedness and enhance their understanding and perception of their roles.	Exercises Self-protection Hygiene Awareness of special disasters (radioactive incidents) Incident command system Lingual skills (multilingualism) Risk control and management Risk relationships
4	Vishnu M. Patel	The study was conducted to find answers to two questions: 1) is the development of an online medicinal curriculum useful for instructing the medical students? 2) Does it cause an increase in their motivations?	American medical university students as volunteers	Fifty-five American medical university students as volunteers	USA	2018	Cross-sectional	Questionnaire before and after the instruction	Four instructional programs with the use of Softchalk software and blackboard learning management system. The students were evaluated using a questionnaire before and after the instruction.	Fifty-five university students participated in this polling. 70% of them expressed that they were not prepared for responding to acute and urgent disasters before the instruction. After the instruction, 11% asserted that they were not ready to do so. 13% of the university students had personal emergency condition kits. 28% had familial disaster-time communication programs. 94% of the students claimed that they completely agreed with the online instructions.	Not mentioned	The online instruction of the volunteers is advantageous in that it needs the lowest resources. The preparation and willingness of the volunteers are increased by these instructions.	Ability in using guidelines Knowledge of disaster terminology Safety kits Triage Emergency planning ICS Surge capacity Ethics Intervention programs for incidents Dangerous and special materials PPEs Decontamination
5	Amanda K. Matthews	The study was carried out with the goal of codifying an instructional program for the volunteers from Vermont’s healthcare department (23) at disaster times.	Nurses from the healthcare department in Vermont, US	3682 nurses from Vermont’s healthcare department participated and 611 completed and returned the questionnaires.	USA	2005	Cross-sectional	Questionnaire before and after the instruction	Instructional program was administered to the nurses within the format of a questionnaire. They were also evaluated before and after the tests.	The main components of the instructional program were an introduction to general hygiene and capacities, an introduction to preparation under emergency conditions, and holding sessions of preparation exercises based on job description papers and guidelines for family and personnel preparation.	Not mentioned	To increase the volunteers’ preparation, holding of multidimensional instructional courses like online instruction, email-based instruction, and person-to-person instruction are very contributive to the effectiveness of the instruction and creation of interest in the learners.	General hygiene Emergency condition planning Communications Information management Epidemiology/healthcare system Regional monitoring
6	Lavonne M. Adams	This study was conducted with the objective of increasing the psychological health nurses’ awareness of the volunteers’ psychological health needs.	Not mentioned	Not mentioned	USA	2007	Review	Not mentioned	Not mentioned	In Rita and Katrina Tornados, the volunteers were found inflicted with psychological side effects like stress, PTSD, insomnia, and suicide thoughts.	Not mentioned	The volunteers have experienced stress frequently and have many psychological health needs. The healthcare and treatment personnel and volunteers should have instructions in line with the enhancement of their psychological health before attending the disaster-stricken regions.	Psychological health
7	Anita Chandra et al.	Evaluation of the knowledge, attitudes, performance, exercises, and psychological first aid (PFA) in a sample of the volunteer members of a medical resources company (MRC) who attended under emergency conditions.	Volunteers of the medical resources company (MRC), US	76 MRC volunteers	USA	2004	Mixed Method	A questionnaire before and after the instruction, centralized group discussions	A questionnaire was given to the volunteers before and after the instruction to complete and centralized group discussions were also dispersed amongst them; hear, protect, and join (PFA model for the individuals) that was concentrated on hearing and understanding the verbal and nonverbal signs, protecting individuals by realistic methods that help increase their self-confidence, and joining individuals in the community. Qualitative data analyses were carried out.	The results of the questionnaire before and after PFA were 71% and 90%, respectively. The results indicated an increase in the individuals’ knowledge after the instruction. Furthermore, these instructions were found influential on reduction of mental stress.	PFA instructions caused an increase in self-confidence, which could influence the test scores.	PFA instructions constitute a method for enhancing the psychological competency of the individuals subjected to disasters.	Steps in PFA are hearing, protecting, and joining Disasters effects Identification of adults’ reactions to disasters Identification of high-risk individuals in disasters Reactions in children
8	Terry Fulmer et al.	The study was conducted to answer the following question: “how may a large private organization participate in and respond to disaster management.	All students from the American universities volunteered.	6000 volunteers from the universities	USA	2007	Cross-sectional	Questionnaire	Two stages of simple randomized sampling were carried out. The questionnaire was administered to the volunteers through the internet. 337 individuals responded to the questionnaires.	The studied data indicated that the private organization’s volunteers tended to provide helps in disasters. Having certain skills, they could also provide more effective responses. 87% of the respondents asserted that family communications were the urgent need of the volunteers.	Not mentioned	The volunteers can participate in preparation exercises for disasters to enhance their performance. It is suggested that the well-trained volunteers should take part in disasters.	Motivational topics Child care Skillfulness in establishing camps and shelters Family communication Adults’ healthcare Leadership
9	Tomoko Haraoka	This study aimed at extracting the factors influencing the cooperation between the earthquake volunteers and victims.	Volunteers of the earthquake in Niigata, Japan	302 volunteers in Niigata, Japan	Japan	2012	Cross-sectional	Questionnaire	From July to September 2008, a questionnaire was given to 302 volunteers in Niigata that was stricken by Nigataken Earthquake in 2007 and the completed questionnaires were returned. Each factor was extracted based on the healthcare opinion model. Multiple regression analysis was utilized.	Out of the 261 questionnaires that had been completed, 41.3% were completed by the leaders who cooperated with the volunteers and 60.2% were completed by the residents who cooperated with the volunteers. The cooperation rate was significantly associated with the earthquake intensity and closeness to earthquake. There was also a significant positive relationship between cooperation and the sense of social solidarity, social capital, and benefits given to individuals.	1. Retrieval error due to the elapse of time from earthquake occurrence 2. Certain individuals had completed the questionnaires 3. Some of the scales had not been tested regarding their reliability and validity.	Cooperation between the volunteers and disaster victims should be carried out before the occurrence of disasters through such measures as evaluation of the region and damages. Moreover, national institutes should provide the local people with information about the contingent earthquake damages. Furthermore, holding instructional and training courses contributes to more cooperation between these groups.	Exercise Emergency Evacuation
10	Anda Kamal	This study aimed to investigate the knowledge and skills of the volunteers responding to disasters.	Published articles that were related to the subject	Twenty-four articles were completely related to the subject	Thailand	2012	Review	Databases and checklists of the articles’ evaluation	Articles published in PubMed, Science Direct, CINHAL, and ProQuest from 2000 to 2011 were investigated.	Twenty-four articles and documents were selected with six components being extracted: 1) initial warning, 2) disaster triage, 3) first aid, 4) search and rescue, 5) logistics and communications, and 6) team organizations.	Not mentioned	The knowledge and skills of the individuals responding to disasters should be increased so that well-trained volunteers can be used on the right time.	Initial warning system Disaster triage Team organizations Search and rescue Logistics and communications
11	Blythe McLennan et al.	This study aimed to express the accomplishments of change in the attitudes towards disaster resilience management by concentrating on Australia.	Not mentioned	Not mentioned	Australia	2016	Review	Not mentioned	Not mentioned	The four primary trends of the volunteers’ roles in disasters in future were: 1) further growth in various kinds of volunteers, 2) effect of communication technology, 3) more interventions in the private sector, and 4) increase in the government’s expectations from the volunteers’ participation. Five areas of opportunity in Australia were: 1) more development of more flexible volunteers in disasters, 2) on-time recalling of the volunteers, 3) increasing the capacity of the digital volunteers, 4) increasing the skills and knowledge of the volunteers, and 5) reducing the disaster risks in the community	Not mentioned	Paying more attention to the presence and empowerment of the volunteers in disasters in Australia can contribute to more coordination of response to disasters.	Use of modern communication technologies Familiarity with the local and national cultures

## Discussion

The present study aimed to extract the instructional titles required by volunteers in disasters of various countries around the globe. According to the results, six cross-sectional studies were dealt with the investigation of knowledge, awareness and instruction of the volunteers through questionnaires. Indeed, two combined articles were dealt with the issue through interviews, modeling methods, questionnaires and focus group sessions. Three review studies were carried out, as well. Out of the extracted studies, six were conducted in the US. After that, the highest studies’ frequency was related to Indonesia, Thailand, Japan, China and Australia since devastative natural disasters had occurred in these countries and volunteers played more accentuated roles in the rescue and relief operations. 

Considering the fact that the society’s resilience is enhanced in the course of disasters, more accentuated roles are expected to be exhibited by the volunteers in disasters. However, the roles of the volunteers have undergone more considerable changes in the 21^st^ century. In Australia, five strategies have been taken into account for emergency conditions management includes the strategies development of flexible volunteers, corroboration of the active and always ready volunteers, surge capacity, digitalization of the volunteers, and growth in the volunteers’ skills and society-oriented interventions to reduce disasters [7]. Certain strategies should be taken into consideration for successful and effective presence of the volunteers in various countries. In this context, digitalization and online management of the volunteers are amongst the most important programs since many of these instructions can be offered through the internet and social networks.

Vermont’s Department of Healthcare (VDH) in the US has codified an instructional program with three goals including background information about general hygiene, preparation under emergency conditions and practical instructions to the volunteers. The main components of the instructional programs are goals and capacities of general hygiene, an introduction to preparation under emergency conditions, exercises, personal and familial preparation exercises and guidelines. In order to increase the volunteers’ potentials, multivariate instructional programs were implemented online, by email, and in person [19]. In this context, the healthcare and treatment instructions are the most essential instructions required by the volunteers because a large number of people who need help will become inflicted with injury and damage.

Initial precaution, disaster triage, search and rescue, logistics and communications, and organizational team are amongst the most important titles that should be taught to the volunteers. However, few studies have been conducted on the knowledge and skill of emergency care in disasters. It has been suggested that the volunteers should be trained in the same way that their capacities can be utilized when there are few or no professionals present on the disasters scene [13]. Start triage should also be taught to the volunteers who pertains to triage in disasters.

Moreover, the volunteers who get present for rescue and relief in disasters should be able to restore themselves psychologically and physically. Furthermore, governmental organizations should provide the damages’ information to predict the disasters course as well as the prevention instruction of disaster occurrence for the local residents. Residents should also prepare the incident map and disaster prevention with the assistance of other habitants. In the end, enhancement of the local associations’ activities might increase the residents’ cooperation [21].

The volunteers’ needs for psychological health in response to disasters are of great importance and should have be taken into account by policymakers. The volunteer’s disaster-responding might have special psychological health needs. Thus, certain measures should be taken into account for psychologically supporting the volunteers who have stress and worries and are not mentally prepared [15]. In this regard, appropriate participation of sophisticated psychologists and psychiatrics can be contributive in disasters.

The volunteers attending disasters should have a good deal of knowledge and awareness; therefore, they can provide proper response to this kind of incidents. Universities are important environments for performing voluntary interventions. A private organization volunteers who are willing to help in the course of natural disasters and have certain skills can also be useful in damages reduction. As for the settlement of the volunteers in the course of disasters, things should be learnt and instructed. The disasters instructing preparation is a rational step for the volunteers in universities [20]. The volunteers’ participation in academic environments before the occurrence of incidents contributes to their higher coordination.

In response to natural and manmade disasters, the governments should offer instructions to improve their performance by considering the daily increasing growth in the number of individuals who taking part. Universities and schools also play determinant roles in this respect. It is hoping that the findings of this study can be effective in codification of an efficient instructional program for enhancing the volunteers’ performance in respond to disasters. 

Considering an effective and decisive presence of volunteers in natural and man-made disasters and their awareness of vital issues such as self-protection with personal protective equipment (PPE), incident command system (ICS) and first aid measures can help save the lives of the injured or sick as was observed in the COVID-19 crisis.

Planners and policy makers of relief organizations includes the Red Crescent, the Red Cross, and the Ministry of Health should formulate a training program to help the integrate maximize training, performance and effectiveness for volunteers. It has reached the ultimate goal of disaster management which is reducing deaths and injuries.

Study Limitation

One of the limitations of this study was using only English made articles. The search was also very extensive and covered a wide spectrum which led to the selection of a large array of articles.

## References

[1] Wang X, Gao L, Shinfuku N, Zhang H, Zhao C, Shen Y (2000). Longitudinal study of earthquake-related PTSD in a randomly selected community sample in north China. Am J Psychiatry.

[2] Organization WH, Retrieved March. 2014 (2013). Building Back Better Sustainable Mental Health Care after Emergencies.

[3] Myhre D, Bajaj S, Fehr L, Kapusta M, Woodley K, Nagji A (2017). Precepting at the time of a natural disaster. Clin Teach.

[4] Benjamin E, Bassily-Marcus AM, Babu E, Silver L, Martin ML (2011). Principles and practice of disaster relief: lessons from Haiti. Mt Sinai J Med.

[5] Australia V (2015). Volunteering Australia Project: The review of the definition of volunteering.

[6] Hockenos P State of the World’s Volunteerism Report: Universal values for global well-being.

[7] McLennan B, Whittaker J, Handmer J (2016). The changing landscape of disaster volunteering: opportunities, responses and gaps in Australia. Natural Hazards.

[8] McDougall K (2011). Using volunteered information to map the Queensland floods. Proceedings of the 2011 Surveying and Spatial Sciences Conference: Innovation in Action: Working Smarter (SSSC 2011).

[9] Haworth B, Bruce E (2015). A review of volunteered geographic information for disaster management. Geography Compass.

[10] Cobb C, McCarthy T, Perkins A, Bharadwaj A, Comis J, Do B (2014). Designing for the deluge: understanding & supporting the distributed, collaborative work of crisis volunteers. Proceedings of the 17th ACM conference on Computer supported cooperative work & social computing.

[11] Yang C-L, Shieh M-C, Huang C-Y, Tung C-P (2018). A Derivation of Factors Influencing the Successful Integration of Corporate Volunteers into Public Flood Disaster Inquiry and Notification Systems. Sustainability.

[12] Yang CL, Shieh MC, Huang CY, Tung CP (2018). A Derivation of Factors Influencing the Successful Integration of Corporate Volunteers into Public Flood Disaster Inquiry and Notification Systems. Sustainability.

[13] Kamal A, Songwathana P, Sia WS (2012). Knowledge and skills of emergency care during disaster for community health volunteers: a literature review. Nurse Media Journal of Nursing.

[14] Khoe LC, Chan EY (2018). Developing Evidence-Based Training Program for Volunteers in Disaster and Emergency Preparedness. Advanced Science Letters.

[15] Adams LM (2007). Mental health needs of disaster volunteers: a plea for awareness. Perspect Psychiatr Care.

[16] Wahyudin D, Hasegawa S, Dahlan T (2013). Developing ethical decision making skill of novice volunteers in natural disaster response. European Conference on Games Based Learning.

[17] Emery RJ, Sprau DD, Morecook RC, Herbold J (2009). Surge capacity volunteer perspectives on a field training exercise specifically designed to emphasize likely roles during a disaster response. Health Phys.

[18] Patel VM, Dahl-Grove D (2018). Disaster Preparedness Medical School Elective: Bridging the Gap Between Volunteer Eagerness and Readiness. Pediatr Emerg Care.

[19] Matthews AK, Sprague K, Girling E, Dapice L, Palumbo MV, Berry P (2005). Emergency preparedness volunteer training program. J Public Health Manag Pract.

[20] Fulmer T, Portelli I, Foltin GL, Zimmerman R, Chachkes E, Goldfrank LR (2007). Organization-based incident management: developing a disaster volunteer role on a university campus. Disaster Manag Response.

[21] Haraoka T, Ojima T, Murata C, Hayasaka S (2012). Factors influencing collaborative activities between non-professional disaster volunteers and victims of earthquake disasters. PLoS One.

[22] Chandra A, Kim J, Pieters HC, Tang J, McCreary M, Schreiber M (2014). Implementing psychological first-aid training for medical reserve corps volunteers. Disaster Med Public Health Prep.

